# Local Melting of Gold Thin Films by Femtosecond Laser-Interference Processing to Generate Nanoparticles on a Source Target

**DOI:** 10.3390/nano8070477

**Published:** 2018-06-28

**Authors:** Yoshiki Nakata, Keiichi Murakawa, Noriaki Miyanaga, Aiko Narazaki, Tatsuya Shoji, Yasuyuki Tsuboi

**Affiliations:** 1 Institute of Laser Engineering, Osaka University, 2-6 Yamadaoka, Suita, Osaka 565-0871, Japan; murakawa-k@ile.osaka-u.ac.jp (K.M.); miyanaga@ilt.or.jp (N.M.); 2 National Institute of Advanced Industrial Science and Technology, Central 5, Higashi 1-1-1, Tsukuba, Ibaraki 305-8565, Japan; narazaki-aiko@aist.go.jp; 3 Graduate School of Science, Osaka City University, 3-3-138 Sugimoto Sumiyoshi-ku, Osaka 558-8585, Japan; t-shoji@sci.osaka-cu.ac.jp (T.S.); twoboys@sci.osaka-cu.ac.jp (Y.T.)

**Keywords:** femtosecond laser, interference, laser processing, melt, nanoparticle, gold, thin film

## Abstract

Shape- and size-controlled metallic nanoparticles are very important due to their wide applicability. Such particles have been fabricated by chemosynthesis, chemical-vapor deposition, and laser processing. Pulsed-laser deposition and laser-induced dot transfer use ejections of molten layers and solid-liquid-solid processes to fabricate nanoparticles with a radius of some tens to hundreds of nm. In these processes, the nanoparticles are collected on an acceptor substrate. In the present experiment, we used laser-interference processing of gold thin films, which deposited nanoparticles directly on the source thin film with a yield ratio. A typical nanoparticle had roundness $f_{r}=0.99$ and circularity $f_{circ}=0.869$, and the radius was controllable between 69 and 188 nm. The smallest radius was 82 nm on average, and the smallest standard deviation was 3 nm. The simplicity, high yield, and ideal features of the nanoparticles produced by this method will broaden the range of applications of nanoparticles in fields such as plasmonics.

## 1. Introduction

Shape- and size-controlled metallic nanoparticles have a variety of applications in the fields of plasmonics, catalysts, biology, etc. Such nanoparticles have been fabricated by chemosynthesis [1,2], chemical-vapor deposition [3], electron-beam lithography [4], laser processing, etc. In laser processing, pulsed-laser deposition (PLD) [5,6,7] and local melting of thin films have been employed, with the latter technique using melting and re-solidification of the film [8,9,10,11]. For area processing by a Gaussian beam, the particle sizes are dispersed due to the Rayleigh instability criterion [8]. On the other hand, localization of the melt area by patterned illumination using a mask to avoid the effects of the Rayleigh instability results in a relatively uniform size distribution [9,10,11]. In this case, the thin film is ablated through the mask, and the rest of the source thin film melts, shrinks, and forms nanoparticles. The laser-interference processing technique has also been applied to metallic thin films. Using this technique, liquid structures such as nanodrops [12,13,14,15], nanobumps, and nanowhiskers [13,14,15,16,17] have been fabricated for cases where the thermal-diffusion length is short when compared to the period of the interference pattern. In these processes, motions of the liquid source metal are induced periodically in space by the interference pattern, and nano-sized structures freeze simultaneously after photon and emission of radiation. We call this mechanism the solid-liquid-solid (SLS) [16], in contrast to the vapor-liquid-solid (VLS) mechanism that is used to generate one-dimensional (1D) nanomaterials [18]. In nanowhisker generation, nanodrops detach from the nanowhiskers and remain on the source target, but they blow off before morphological measurements can be carried out. In some other experiments, a single-focus spot has been employed in the laser-induced dot transfer (LIDT) technique [19,20,21,22]. This is a variation of the laser-induced forward transfer (LIFT) technique [23,24,25] where a nanoparticle from a source film is caught on an acceptor substrate.

In the present experiment, we collected gold nanoparticles directly on the source target without requiring an acceptor substrate (Laser-Induced Dot caught On Source target; LIDOS). We measured the morphologies of the nanoparticles using scanning electron microscopy (SEM) without any cleaning of the target. We analyzed the dependence of the size distributions of the nanoparticles on parameters such as the film thickness and fluence. The results that we obtained are supported by a model that explains the formation mechanism.

## 2. Materials and Methods

**Figure 1:**
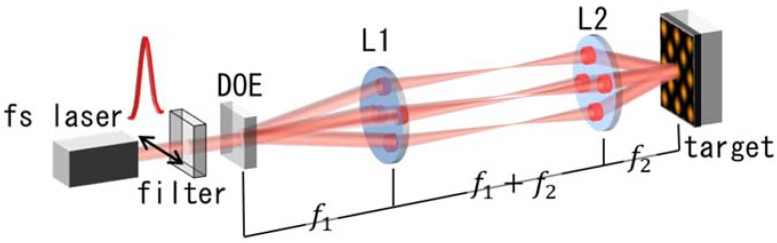
Experimental setup. The pulse width of the laser was 240 fs and the wavelength was 785 nm. DOE (diffractive-optical element) split four 1st order diffracted beams. $f_{1}=300$ mm and $f_{2}=40$ mm.

Our experimental setup is shown in Figure 1. We used a femtosecond laser beam (IFRIT, Cyber Laser, Tokyo, Japan) operating at 785 nm with a 240 fs (full-width at half-maximum, FWHM) pulse width. The beam was split by a diffractive optical element, which was optimized to the wavelength (the diffraction efficiency was approximately 60%) to generate four first-order diffracted beams. The four beams were focused on the surface of the target via a demagnification system, for which the focal lengths of the achromatic lenses were $f_{1}=300$ mm and $f_{2}=40$ mm. The original beam diameter was 6.0 mm (FWHM at $1/e^{2})$, which was de-magnified to 0.8 mm. The zeroth-order beam was dumped. The correlation angle was θ=16.7°, and the period of the interference pattern was Λ=19.3 μm. For this experiment, we chose a gold thin-film target, which has surface-plasmon resonance [26] and stability. We deposited the films on silica-glass substrates by magnetron sputtering and was electrically insulated from the earth. All of the experiments were performed using a single shot of laser irradiation at atmospheric conditions. We imaged the surface structures of the films using SEM (JEOL, JSM-7400FS, Tokyo, Japan).

## 3. Results and Discussion

**Figure 2:**
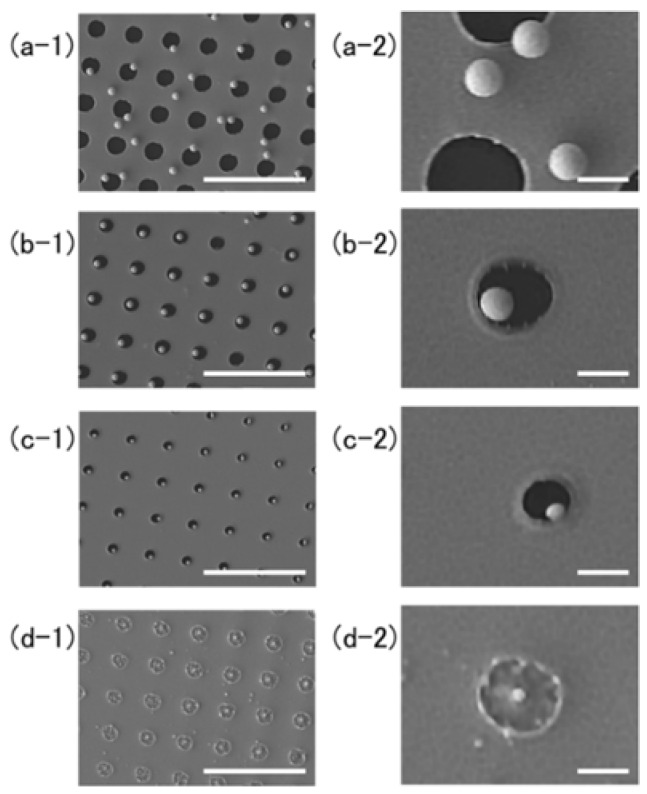
Scanning electron microscopy (SEM) images of the target surface with different film thickness. (**a-1**) 40 nm; (**b-1**) 50 nm; (**c-1**) 60 nm; and (**d-1**) 100 nm, and their magnified images on right column: (**a-2**) 40 nm; (**b-2**) 50 nm; (**c-2**) 60 nm; and (**d-2**) 100 nm. The experimental parameters and results are summarized in Table 1. The white bars in the images on the left and right columns represent 5 μm and 500 nm, respectively.

**Figure 3:**
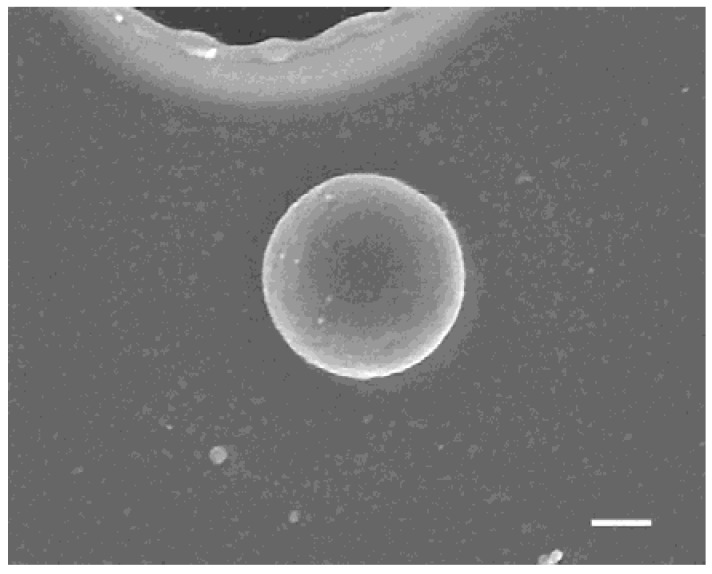
Observation with high-resolution scanning electron microscopy (HR-SEM) of a single nanoparticle shown in Figure 2a-1. The film thickness was 40 nm, and the fluence was 73.2 mJ/cm^2^. The radius was 175 nm. The white bar in the image represent 100 nm.

Figure 2 shows a top view of the structures fabricated with different film thickness. The target-film thickness, the average fluence over the interference pattern, the average radius and its standard deviation (s.d.) are summarized in Table 1. The precise lattice structures with Λ=19.3 μm period were determined by the interference pattern. For the thinner film of 40 nm thickness, the nanoparticles were found to lie on the source target at random locations, as shown in Figure 2a-1. The circular black holes are the areas from which the particles were ejected and through which the substrate surface appeared. Figure 2a-2 is a magnified view of Figure 2a-2, and demonstrates that particles with good roundness could be fabricated. The average radius of the nanoparticles was 188 nm, and the standard deviation was 7 nm. The yield ratio, which is the number of nanoparticles relative to the number of holes, was 83% in the field of view in Figure 2a-1. Figure 3 shows a high-resolution SEM (HR-SEM) image of the same field with 100,000× magnification. The surface of the nanoparticle was smooth, and some nanostructures smaller than 10 nm adhered to the surface; these may have been smaller nanoparticles that had condensed from the gold vapor. The radius of this nanoparticle was 174.8 nm. The circularity $f_{circ}$ and roundness $f_{r}$ of the nanoparticle were defined by the following equations:

(1) $$f_{circ}=4\pi S/P^{2}$$

(2) $$f_{r}=4S/{\left(2a\right)}^{2},$$

where S is the surface area of the nanoparticle; P is the perimeter; and 2a is the length of the major axis, assuming the shape to be an ellipsoid. For this nanoparticle, we obtained $f_{circ}=0.869$ and $f_{r}=0.99$, so it was a fairly round sphere with a slightly rough surface. Note that $f_{circ}$ is affected by the resolution of the SEM.

When the film thickness was 50 nm, smaller nanoparticles were fabricated, as shown in Figure 2(b-1,b-2). Most remained on the lower left sides of the holes, but the reason for this is not known. The yield ratio was 93%. The standard deviation of the radius was 3 nm, which was the smallest dispersion in this experiment. When the film thickness was 60 nm, smaller nanoparticles always remained on the lower right edges of the holes, as shown in Figure 2(c-1,c-2). The yield ratio of the field of view was 100%. When the thickest film of 100 nm was used, only the textured surface of the gold film was seen, as shown in Figure 2(d-1,d-2). White spots, which show the existence of projecting structures, could be seen at the centers of the depressions in the processed area. Owing to the thickness of this film, none of these depressions punched through to expose the substrate. 

**Figure 4:**
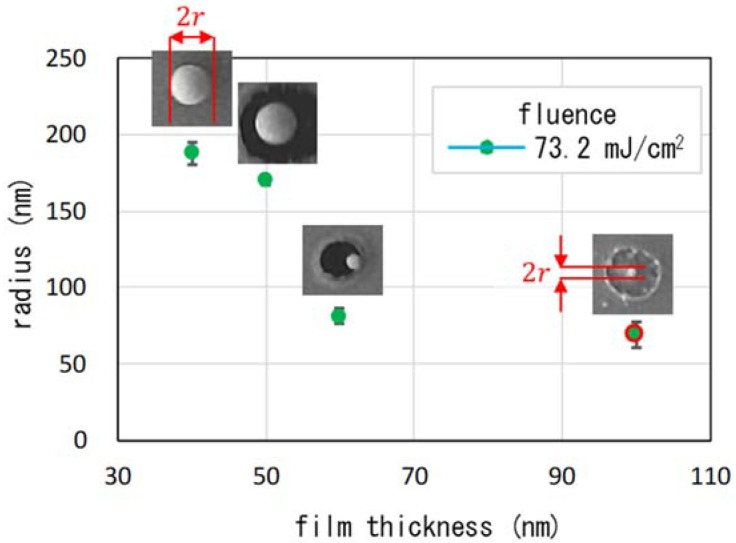
The radii of the nanoparticles as a function of film thickness. The fluence was 73.2 mJ/cm^2^. The error bars reflect the s.d.

**Figure 5:**
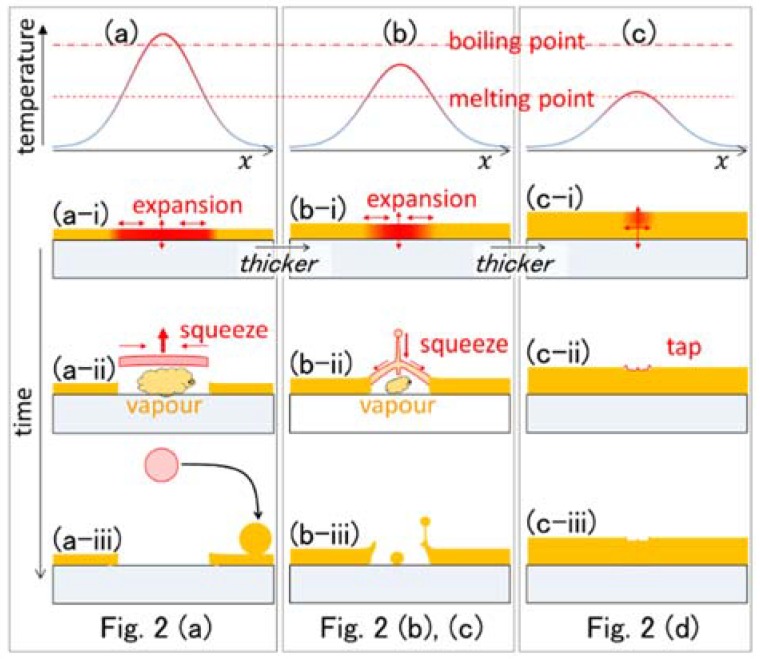
Schematic illustration of the solid-liquid-solid (SLS) process. Each panel illustrates the temporal change of a spot in an interference pattern. The correspondence with the results shown in Figure 2 is noted on the bottom. From (**a**–**c**), the thickness of the film is listed as following: (**a**), 40 nm; (**b**), 50 and 60 nm; (**c**), 100 nm.

**Figure 6:**
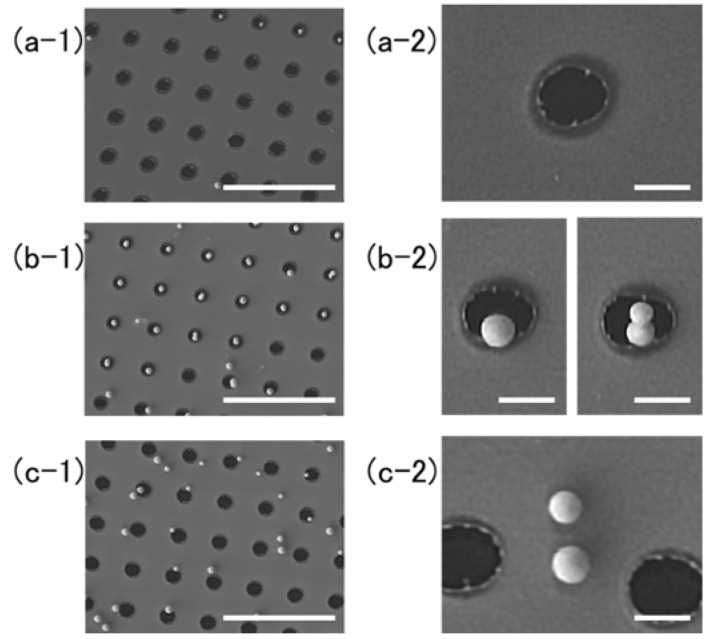
SEM images of the target surface. (**a-1**) film thickness was 30 nm, and fluence was 58.6 mJ/cm^2^; (**b-1**) film thickness was 40 nm, and fluence was 58.6 mJ/cm^2^; (**c-1**) film thickness was 40 nm, and fluence was 169.9 mJ/cm^2^. Pictures on the right column are the corresponding magnified images: (**a-2**) film thickness was 30 nm, and fluence was 58.6 mJ/cm^2^; (**b-2**) film thickness was 40 nm, and fluence was 58.6 mJ/cm^2^; (**c-2**) film thickness was 40 nm, and fluence was 169.9 mJ/cm^2^. The experimental parameters and results are summarized in Table 1. The white bars in the images on the left and right columns represent 5 μm and 500 nm, respectively.

The radius as a function of film thickness is summarized in Figure 4. The smallest film thickness was 40 nm. The radius of the nanoparticles was smaller than 200 nm in all cases. It is apparent that the radius of the nanoparticles decreased as the film thickness increased from 40 to 60 nm. The results that we obtained are supported by a model that explains the formation mechanism, as shown in Figure 5 [16,27,28]. For every thickness, the films were almost opaque at 785 nm. As the thermal conductivity of gold at room temperature is far higher than that of silica glass, the temperature distribution along the substrate is normal inside the gold film levels immediately. As a result, the rise in temperature is inversely proportional to the mass of the film, as illustrated in the top curves in the figure. For the thinner film shown in Figure 5a, the region at temperatures above the melting point ($T_{m.p.}=1064\;{}^{\circ}C$) was wider than that for the thicker films, as illustrated in Figure 5b,c. The molten layer was launched from the substrate by the reaction to volumetric expansion during the solid-to-liquid phase transition, as shown in Figure 5a-i. It was then squeezed and formed a nanoparticle due to surface tension, as in Figure 5a-ii. It solidified because of cooling by photon emission, and could then be pulled by electrostatic forces and adhered to the target surface, as shown in Figure 5a-iii. Most of the nanoparticles adhered to the metal surface because of the metallic bond. However, when the film was too thin, as shown in Figure 6(a-1,a-2), the region simply boiled away.

For the film thickness of 50 nm, the temperature was lower, which resulted in a less-effective launch, as shown in Figure 5b-i. A nanoparticle is formed at the top center of the molten layer because of surface tension, as illustrated in Figure 5b-ii. If it freezes at this time, it can form a nanodrop on a hollow bump [12], or a nanowhisker [16,17], which is left after the nanoparticle detaches. The resulting nanoparticles are left on the processed area or squeezed to the edges of the holes by surface tension, as illustrated in Figure 5b-iii and shown in Figure 2b,c. For a film thickness of 100 nm, a hole could not be punched through the gold film at the given fluence, as shown in Figure 2(d-1,d-2). In this case, the motion of the molten layer resulted in a textured film surface, as shown in Figure 5c-i–c-iii.

**Figure 7:**
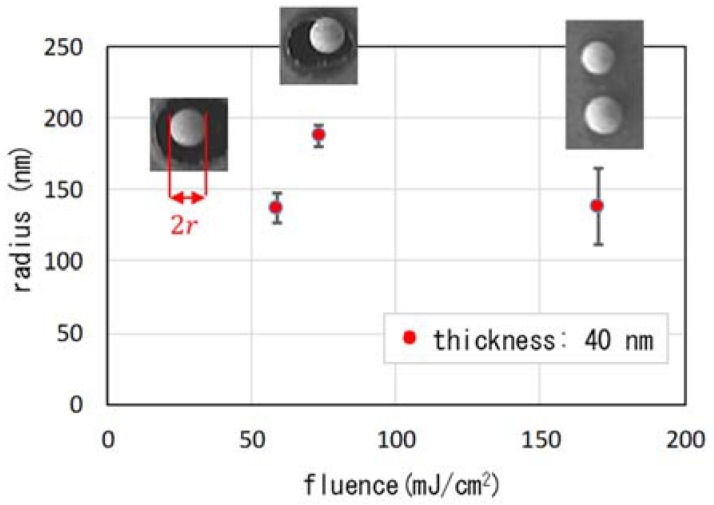
Radius of nanoparticles as a function of fluence. The film thickness was 40 nm. The error bars reflect the s.d.

The radius of the nanoparticles as a function of the fluence are summarized in Figure 7 for a film thickness of 40 nm. The radius was smaller than 200 nm in all cases. It is interesting that mixed nanoparticles such as singles and twins were produced, as shown in Figure 6(b-1,b-2). In Figure 7, data for single particles are plotted. It is interesting that the formation of twins or multiple droplets in a string is seen also in the behavior of water solutions [29]. The process of drop detachment was controlled in those experiments by dissolving polymeric molecules, thus changing the viscoelasticity of the solution. In our case, it may be possible to control multiple-nanoparticle formation by changing the material, e.g., using silver, chromium and alloys to obtain different values of viscoelasticity. When the fluence was at the highest value that we employed, 169.9 mJ/cm^2^, the process caused more spattering. As can be seen in Figure 6(c-1,c-2), the standard deviation of the radius was highest, 27 nm. 

We now compare this method with others. The roundness of the nanoparticles produced in this experiment was excellent, and the radius controllable between 69 and 188 nm. In PLD, the radii of the nanoparticles that condensed from the gas phase ranged from a few nm to some tens of nm [5,6]. On the other hand, the droplets fabricated by PLD were some hundreds of nm in radius [7]. Using LIDT, nanoparticles with radii smaller than 250 nm can be fabricated [19,30]. Electron-beam lithography is utilized for fabricating aligned nanoparticles a few nm in diameter [4]. Chemosynthesis can be used to fabricate nanoparticles and nanorods with radii of a few nm in both structures [1]. In summary, this technique is a good alternative to PLD or LIDT, and can generate nanoparticles with radii of tens or hundreds of nm via the SLS process.

## 4. Conclusions

Using the SLS process, we successfully fabricated gold nanoparticles with radii of tens to hundreds of nm and good roundness-which were subsequently deposited on the source substrate-using irradiation through the interference pattern of a fs laser. The film thickness and fluence were the key parameters for controlling the sizes of the nanoparticles, with a thinner film resulting in a larger nanoparticle radius. The smallest radius was 82 nm on average, and the smallest standard deviation was 3 nm. A typical nanoparticle was found to have roundness $f_{r}=0.99$.

Compared with the methods such as chemosynthesis, VLS and PLD, SLS is useful for fabricating pure and uniform nanomaterials. No catalyst or chemosynthetic solution is required, and a more uniform size distribution will result in better plasmonic resonance properties. We anticipate that different source materials such as metals, alloys, and non-metals with plasticity can produce nanoparticles using the SLS mechanism. The process is very simple and does not require cleaning, temperature control, evacuation, purification, etc. These advantages will broaden the range of applications for such nanoparticles. 

## Figures and Tables

**Table 1:** Parameters and results for the experiments shown in Figure 2, Figure 3, and Figure 6. In the case of Figure 2c, nanoparticles should adhere to the edge of holes of the film and not be separated. In the case of Figure 2d, the nano-structure was nano-projecting, not nanoparticle.

Parameter	Figure 2(a-1,a-2), Figure 3	Figure 2(b-1,b-2)	Figure 2(c-1,c-2)	Figure 2(d-1,d-2)	Figure 6(a-1,a-2)	Figure 6(b-1,b-2)	Figure 6(c-1,c-2)
film thickness (nm)	40	50	60	100	30	40	40
fluence (mJ/cm^2^)	73.2	73.2	73.2	73.2	58.6	58.6	169.9
averaged radius (nm)	188	170	82	69	n.d.	137	138
standard d. (nm)	7	3	5	9	n.d.	10	27
